# Lifestyle or pharmacotherapy in cardio-metabolic disease prevention

**DOI:** 10.1177/17539447231177175

**Published:** 2023-06-29

**Authors:** Borenyi S. Seidu, Hanad Osman, Samuel Seidu

**Affiliations:** The University of Manchester, Manchester, UK; Leicester Diabetes Centre, Leicester General Hospital, Leicester, UK; Diabetes Research Centre, University of Leicester, Leicester General Hospital, Leicester, UK; Diabetes Research Centre, University of Leicester, Leicester General Hospital, Gwendolen Road, Leicester LE5 4WP, UK Leicester Diabetes Centre, Leicester General Hospital, Leicester, UK

**Keywords:** cardio-metabolic disease, lifestyle, multimorbidity, pharmacotherapy, prevention

## Abstract

Cardio-metabolic diseases are the leading causes of premature death worldwide. The conditions are together some of the most prevalent and severe multimorbidities and include conditions such as diabetes, hypertension, coronary heart disease and stroke. People with these conditions are at a higher risk of all-cause death and have a reduction in life expectancy when compared to patients without cardio-metabolic disorders. As a result of the increasing prevalence and impact of cardio-metabolic multimorbidity on disability, no healthcare system can ‘treat’ its way out of this pandemic. ‘Treating our way out’ requires the use of multiple medications which can lead to improper prescribing, insufficient compliance, overdosing or underdosing, improper drug choice, insufficient monitoring, unfavourable drug effects, and drug interactions and inappropriate wastes and costs. Therefore, individuals living with these conditions should be empowered to adopt lifestyle changes that foster independent living with their conditions. Adopting these healthy lifestyles such as smoking cessation, improving dietary habits, sleep hygiene and physical activity is a suitable adjunctive measure if not an alternative to polypharmacy in cardio-metabolic multimorbidity.

## Background

Cardio-metabolic diseases (CMDs) include many linked conditions including type 2 diabetes, hypertension, stroke, dyslipidaemia and other cardiovascular diseases (CVDs).^1^ CMD is one of the leading causes of premature death worldwide. It is estimated to have caused 18 million deaths worldwide in 2019.^2^ In the United Kingdom there are approximately 8 million people living with CMD.^3^ Some of the key significant risk factors for CMD include poor dietary habits, physical inactivity, smoking and sleep deprivation.^4–6^

Diabetes is a leading cause of many complications including heart attacks, kidney failure, stroke, loss of sight and amputation of lower limbs.^7^ There is a two to three times increased risk of strokes and heart attacks in adults with diabetes.^8^ Due to neuropathy, people with diabetes also have an increased risk of foot ulceration which can lead to infection and can also possibly result in the need for amputation of the limb.^9^ Damage to the small blood vessels in the retina over a long period of time can lead to diabetic retinopathy. This is a major cause of blindness and it is estimated that nearly 1 million people have lost their sight due to diabetes. Diabetes has also been linked with worse outcomes for COVID-19 and other infectious diseases.^7^

It is estimated that diabetes and kidney disease caused by diabetes were responsible for the deaths of around 2 million people worldwide.^7^ The global incidence of diabetes in adults aged between 20 and 79 years is growing. In 2021, it was estimated that there were 537 million people living with diabetes which would have equated to approximately 10% of the global population. This is predicted to increase to 643 million people in 2030, and in 2045 it is predicted to increase even further to 783 million people. In Europe, 7.7% of all deaths occurring before the age of 60 are due to diabetes.^10^

In the United Kingdom there are around 5 million people living with diabetes with around 4 million adults diagnosed with the condition. There are nearly 1 million people in the United Kingdom living with undiagnosed type 2 diabetes. Around a third of adults in the United Kingdom living with diabetes will die from a heart or circulatory disease. Overall adults living with diabetes are nearly twice as likely to die from stroke or heart disease compared to adults that do not have diabetes.^3^

Diabetes has a significant cost to the UK health economy. The net ingredient cost of insulin items prescribed in England between 2012 and 2013 was £320 million. This represents an increase of £100 million since 2005–2006.^11^ Furthermore there was a seven-fold increase in the number of people with type 2 diabetes using insulin in the United Kingdom between 1991 and 2010. This rise is likely to be linked to better diagnosis rates, increasing incidence, longer survival and changes in the management of type 2 diabetes.^12^

Hypertension is another condition that is heavily linked with serious consequences. It is estimated that hypertension is accountable for approximately 50% of strokes and heart disease.^13^ Globally in 2010 it was estimated that there were nearly 1.4 billion people affected by hypertension which equated to around 30% of the world’s population.^14^ It is thought that this will increase to just under 1.6 billion people in 2025.^15^ There are a few factors that are thought to be responsible for the increasing prevalence of hypertension. Some of the factors include people living longer and an increase in worsening lifestyle choices such as diets high in salts and a more sedentary lifestyle.^14^

In the United Kingdom it is estimated that there around 15 million people living with hypertension which equates to 28% of the adult population. There is around 10 million people with a diagnosis of hypertension which leaves around 5 million people that are not aware that they may have hypertension. It is estimated that in total that there are up to 8 million people that are living with undiagnosed or uncontrolled hypertension.^3^

Multimorbidity is defined as an individual living with two or more long-term health conditions. Long-term health conditions can include a variety of conditions such as those that impact physical health and mental health.^16^ One retrospective cohort study in England found that the prevalence of multimorbidity was just over one in four. This study also found that that there was a significant association between increased age and prevalence of multimorbidity.^17^

Overall, there is a large number of people living with multiple conditions and the prevalence of multimorbidity is increasing in the older population.^18^ It is predicted that the prevalence of multimorbidity will continue to increase over the next couple of decades. A study has predicted that the percentage of people over the age of 65 living with two or more health conditions will increase from 54% in 2015 to 68% in 2035. This study also predicted that the prevalence of people living with four or more health conditions will double to 17% in 2035 which would equate to around 2.5 million people.^19^

One of the most prevalent and severe multimorbidities is cardio-metabolic multimorbidity (CMM), which is defined as the presence of two or more cardio-metabolic disorders, such as diabetes, hypertension, coronary heart disease (CHD) and stroke.^20–22^ People with CMM had a 3.7–6.9 times higher risk of all-cause death and a 12–15 year reduction in life expectancy at age 60 when compared to patients without cardio-metabolic disorders.^20^

As a result of the increasing prevalence and impact of CMM on disability and mortality, quality of life and increased disease load, reliance on healthcare systems to tackle this worsening problem will place significant strain on most health economies. Individuals living with these conditions should therefore be empowered to adopt lifestyle changes that foster independent living. Unfortunately, in most healthcare systems, there is still the over-reliance on pharmacotherapy to treat these cardio-metabolic long-term conditions. Even though a lot of these medications are based on evidence for the outcomes they are intended to address, their use in addressing CMM is often fraught with improper prescribing, insufficient compliance, overdosing or underdosing, improper drug choice, insufficient monitoring, unfavourable drug effects, and drug interactions and inappropriate wastes and costs.

## Demerits of pharmacotherapy in CMM

To improve life expectancy or quality of life for long-term conditions, multiple drugs may need to be prescribed to the patient and taken regularly. Although there is no strict definition, polypharmacy usually refers to the usage of multiple drugs, generally seen in the older population due to the decrease of general health along with the effects of ageing.^23^ Over time, both life expectancy and population size have increased which means more of the population live longer with chronic diseases and therefore results in polypharmacy becoming increasingly more common.^24^ Polypharmacy is becoming more of a problem as it leads to an increased risk of inappropriate prescribing of medications, increased chance of adverse effects and other problems relating to drug adherence.^25^

The PRACtICe study in the United Kingdom commissioned by the General Medical Council showed that there may be safety concerns with polypharmacy. The study looked at General Practice prescribing errors and found that for one added medication, the chance of an error occurring related to it rose by 16%. On average, over two-third of patients who are on over nine medications risk having severe drug interactions.^26^

There are other factors healthcare professionals may need to consider when potentially prescribing multiple medications. Adherence to regimes can increasingly become more difficult especially with multiple medications. This can be unintentional such as forgetting to take a dose or intentional due to the patient’s own preference, thinking they can afford to skip a dose, that a medication may be unimportant, that side-effects may be unbearable or they dislike the medication due to palatability.

Data on medication adherence may be overestimated by up to 200% according to a study due to the preferred requirement that patients should self-report.^27^ Despite this, we can see visible patterns from data taken on adherence rates. In a survey, 128 patients diagnosed with diabetes took an average of 4.1 (±1.9) medications to control it among other related issues. This totalled to 523 medicines with 5.8 medicines on average taken daily when considering all medications including prescriptions and over-the-counter medicines. Patients had problems with 10% of the 523 medications with 58% of those medications causing uncomfortable side-effects, 23% being missed due to forgetfulness and 8% were considered expensive.^28^

Medicine non-adherence however becomes more evident in patients being treated for CMD and hypertension. A study looking at this found that 43% of patients were non-adherent to their CMD medications.^29^ This can result in an increased mortality risk of between 50% and 80% for patients with CMD.^30^

Medication costs also play a part in non-adherence. A US-based study which used the Truven Health MarketScan^®^ Research databases between 2011 and 2015 sampled adults with type 2 diabetes on basal insulin therapy. Among the 21,363 sampled, 33.8% of the patients were adherent to their basal insulin therapy and as a result suffered much higher medication-related costs.^31^ Though adhering to drug regimens is helpful, some patients cannot keep up with the costs and, as a result, prefer not to adhere in order to save money for themselves.

Costs of treatment and medication have also been shown to have a burden for the National Health Service (NHS). A study done by the English Longitudinal Study of Ageing found that the CMD incidence has slowed down and started to plateau. If this plateau is to continue, this could roughly cost £54 billion.^32^ Therefore, it is likely polypharmacy will continue to have an impact and lead to a continuing increase in the cost to the healthcare system.

Additionally, a population-based study in Finland between the start of 1995 and the end of 2007 observed the effects of patients taking regular antihypertensive medications after 2 and 10 years. The study found that non-adherent patients were three to four times at risk of dying from a stroke.^33^ The data demonstrate the huge risks associated with medication non-adherence, especially in patients being treated for CMD.

Overall, polypharmacy is increasingly difficult to keep up with over time especially in patients with long-term conditions. With the elderly population growing, the prevalence of polypharmacy will only increase to manage long-term conditions. This has caused healthcare professionals to consider alternate treatments for chronic diseases that do not require any kind of treatment with drugs and instead focus on changing behaviours.^24^

## Merits of lifestyle interventions in the management of CMM

CMD can be directly caused by a poor diet, reduced physical activity, poor sleep patterns and smoking; and addressing these risk factors can foster patient independence and can reduce the need for multiple medications. Evidence-based dietary options known to help prevent CMD include the Mediterranean diet, vegetarian or vegan diets, a portfolio diet and a Nordic diet plan.^34^

The Mediterranean diet, consisting of fruits and vegetables, fish, legumes and nuts, wholegrains and monosaturated fats from olive oils, is shown to greatly help protect against CMD and other illnesses and diseases such as depression and asthma, sometimes even preventing them entirely.^35^ An analysis using the Markov model on cost utility found that the Mediterranean diet costs AU$1013 per quality-adjusted life year gained per person when compared with the Western diet and this caused a mean gain of 0.31 life years per person when it came to life expectancy.^36^ This can be covered by the NHS as it falls in the £20,000–£30,000 threshold and is much more cost-effective than most drugs.^37^ The effect of diet was observed during a study and showed that when the Mediterranean diet was followed more strictly, the risk of developing CMD was reduced.^38^ Similarly, the PREDIMED trial was a randomized controlled trial that looked at the effects of a Mediterranean diet on CVD risk in over 7000 individuals. The study found that the Mediterranean diet reduced the risk of cardiovascular events by 30% compared to a low-fat diet, without the need for medication.^39^

According to Kahleova *et al.*, the vegetarian diet is highly effective for combating CHD mortality and the risk of developing CMD as it can reduce them both by 40%. This diet also has the potential to reverse CHD and is said to be the only diet that can achieve this along with other plant-based diets.^40^ Vegetarian diets are also said to be best for treatment and prevention of type 2 diabetes when compared to other medications.^41^

The portfolio diet is said to help with CMD by lowering cholesterol in the body. This is done through the intake of foods such as soluble fibres, tree nuts, soy proteins and plant sterols.^42^ A study by Chiavaroli *et al*. saw the effect of the portfolio diet on low-density lipoprotein cholesterol (LDLC). When combined with the National Cholesterol Education Program Step II diet, LDLC was reduced by around 17% and so reduced the 10-year risk of CHD. The portfolio diet has been shown to be effective also for patients to follow instead of taking many potential medications which have higher risks for the same reward.^43^

LDLC is also reduced by the Nordic diet, which is very similar to the Mediterranean diet, the difference being that the Nordic diet favours canola oil over olive oils.^44^ A study observed the LDLC levels drop by 21% as well as a reduction in blood pressure. This reduces the risk profile overall for patients at risk of CMD in the first place.^45^ The Okinawan-based Nordic diet has also been shown to be very effective by improving the homeostasis of glucose over 12 weeks which directly helps reduce the risk of developing type 2 diabetes.^46^ Together, these diets can help people consume less than 300 mg of cholesterol daily and ensure that their saturated fats intake is ⩽7% of total energy intake and that their total fat intake is ⩽30% of total energy intake which is recommended for the prevention of CMD.^47^

Along with diets, some commonly used nutritional supplements that have been studied for their potential cardiovascular benefits include omega-3 fatty acids, vitamin D, magnesium and Coenzyme Q10. Omega-3 fatty acids, found in fish oil supplements, have been shown to have modest reductions in cardiovascular events, particularly in people with high triglyceride levels.^48^ Vitamin D supplementation has been associated with improved endothelial function, lower blood pressure and reduced inflammation, but there is still much debate about the optimal level of vitamin D intake and the potential risks of over-supplementation.^49^ Magnesium supplementation has been shown to lower blood pressure and improve insulin sensitivity in some studies, although evidence is not consistent across all studies.^50^

Recent evidence suggests that the gut microbiota and their metabolite products, including short-chain fatty acids (SCFAs), bile acids and amino acids, may play a significant role in the development of CMDs such as obesity, type 2 diabetes and CVD. One review article highlights the role of gut microbiota in the pathogenesis of CMD. The review suggests that changes in the gut microbiota composition, diversity and function, also known as dysbiosis, can lead to the development of insulin resistance, inflammation and oxidative stress.^51^ This review article also focuses on the role of SCFAs in CMD. SCFAs are the primary products of bacterial fermentation of dietary fibres in the gut, and they have been shown to have beneficial effects on host metabolism by improving insulin sensitivity, reducing inflammation and promoting energy expenditure. However, the review notes that the exact mechanisms by which SCFAs exert their effects on host metabolism are not fully understood.^51^

Alongside diet, exercise is well supported and encouraged to help prevent CMD. Physical activity is said to prevent many of the factors causing CMD. Exercising 30 min, five times a week is recommended by the American Heart Association to reduce the risk of developing CMD.^52^ Different types of exercise can play a variety of roles in CMD prevention as well. The effects of aerobic exercises, resistance training and both combined can be noted within 8 weeks.^53^ These exercises were done 3 days a week for an hour, the combined training split into 30 min each. After 8 weeks, aerobic training reduced the mass of fat and body weight as well as increasing cardio-respiratory fitness. Resistance training was found to decrease the waist circumference and increase lower body strength. When combined, they had the best effect, reducing blood pressure, increasing both upper and lower body strength and increasing lean body mass.^53^

A study by Naci and Ioannidis^54^ suggests that drugs and exercise may be just as effective as each other, except for stroke rehabilitation where exercise seemed to prevail and CHD where medication was often the better option. If this is the case, patients may be encouraged to do an hour of exercise regularly to see positive changes in their health as the benefits are comparable to medications but without the risk of potential side-effects.

Another lifestyle change that has the potential to prevent and reverse CMD is the quantity and quality of sleep the patient has. An observational study showed that a shorter sleep pattern influenced by genetics was a potential risk factor for CMD whereas this was unlikely the case for genetically longer sleep patterns.^55^ Insomnia may also lead to an increase of multiple different CMDs morbidity and mortality risks, demonstrated by multiple studies over the last 10 years.^56^ Another study associated both long and short sleep cycles with an increased risk of developing CMD and potentially death.^57^ Overall, a healthy 7–8 h is recommended as the best sleeping pattern to ensure the best outcome for adults as insufficient sleep can increase the risk of developing heart disease as well as an increased risk of developing obesity and diabetes. Maintaining regular sleep patterns, with good quality of sleep as well as quantity is optimal for prevention of heart diseases, being potentially as important as diet and exercise in CMD prevention.^58^

Smoking continues to contribute significantly to annual death and morbidity, mostly due to cancer, vascular and pulmonary conditions.^59–61^ Despite overwhelming proof of the risks of smoking and the advantages of quitting, millions of adults still consume cigarettes.^62^ The benefits of reducing CMD risk from quitting smoking grow with the amount of time since quitting.^62^ In one study, participants who used nicotine replacement therapy or e-cigarettes with or without nicotine had improved cardiovascular health prospects. Both generally speaking and in individuals who successfully stop, these gains can be seen in changes in endothelial function, which occur fairly early in the timeline of smoking cessation.^63,64^

When combined with a healthy diet and regular aerobic and resistance training, improvements in sleep hygiene and smoking cessation, the use of multiple medications may be minimized, reducing polypharmacy and the risk of developing adverse effects, ensuring patients are treated safely as well as benefitting from improvements in their quality of lives and also reducing the burden on healthcare professionals and the wider health system.

Overall lifestyle modifications, such as changes in diet, exercise and stress management, have been shown to have significant impacts on health outcomes and can reduce or eliminate the need for medication in certain populations. The Diabetes Prevention Program found that a structured lifestyle intervention was more effective than medication in preventing the development of type 2 diabetes. This was a large clinical trial that involved over 3000 participants with prediabetes. The study found that a structured lifestyle intervention that included dietary changes and increased physical activity was more effective than medication (metformin) in preventing the development of type 2 diabetes. Participants in the lifestyle group experienced a 58% reduction in the incidence of diabetes, while those in the medication group had a 31% reduction.^65^

Implementing lifestyle modifications can be challenging, as individuals may face barriers such as lack of knowledge, skills, motivation or social support. Thus, it is important to provide not only simple suggestions, but also more comprehensive and personalized approaches that address the specific needs and preferences of each individual. Specialized teams, such as registered dietitians, exercise physiologists, behavioural psychologists and health coaches, can play a crucial role in facilitating lifestyle changes and improving health outcomes. For example, the Diabetes Prevention Program implemented a lifestyle intervention with the help of trained lifestyle coaches who provided individualized feedback, goal-setting and support for dietary and physical activity changes.^65^ By using a team-based approach, lifestyle modifications can become more feasible, sustainable and effective for individuals who seek to improve their health and reduce medication use.

**Figure:**
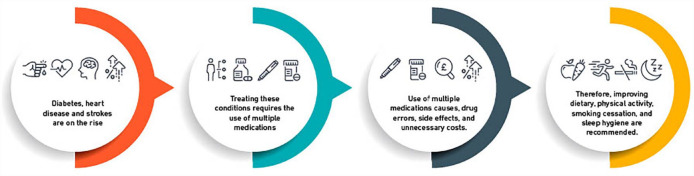
Schematic representation of how lifestyle measure could be the solution to the problems associated with polypharmacy in cardiometabolic multimorbidities.

While lifestyle interventions have shown promising results in improving health outcomes and reducing medication use, there are several challenges that need to be addressed to enhance their effectiveness and sustainability. One of the main challenges is high dropout rates. Many studies have reported that a significant proportion of participants discontinue or fail to adhere to lifestyle interventions, often due to factors such as time constraints, lack of social support, competing priorities or relapse of unhealthy habits. In the Diabetes Prevention Program, 26% of participants did not meet the goal of at least 150 min of physical activity each week at the 24-week stage. At the most recent visit that proportion increased to 42% of participants.^66^

While lifestyle modifications can be a powerful tool for improving health outcomes, medication is still necessary for many people with chronic conditions, and lifestyle modifications should not be seen as a replacement for appropriate medical care.

## Conclusion

CMD tend to be managed with multiple medications, especially in older patients which may increase the risk of further health issues. Drug interactions and non-adherence can lead to further progression of the disease state and need of further therapeutic treatment. When treating patients, clinicians and healthcare professionals want the best possible care for the patient which can maintain or elevate their quality of life. This is where lifestyle changes can play a role and thus reducing the need for polypharmacy. This includes measures such as exercise, particularly aerobic exercise combined with resistance training, smoking cessation and a balanced diet. Quality of sleep as well as the length of sleep should be highly encouraged as it can play a big part in protecting against CMD. When combined, lifestyle changes may be just as effective as pharmacotherapy but reduces the risks of complications and side-effects as well as costs, making it potentially a better treatment for CMD.

## Declarations

**Ethics approval and consent to participate:** Not applicable.

**Consent for publication:** Not applicable.

**Availability of data and materials:** Not applicable.
